# Safety of Low-Dose Aspirin in Endovascular Treatment for Intracranial Atherosclerotic Stenosis

**DOI:** 10.1371/journal.pone.0105252

**Published:** 2014-08-21

**Authors:** Ning Ma, Ziqi Xu, Dapeng Mo, Feng Gao, Kun Gao, Xuan Sun, Xiaotong Xu, Lian Liu, Ligang Song, Tiejun Wang, Xingquan Zhao, Yilong Wang, Yongjun Wang, Zhongrong Miao

**Affiliations:** 1 Department of Interventional Neuroradiology, Beijing Tiantan Hospital, Capital Medical University, Beijing, China; 2 Department of Neurology, Beijing Tiantan Hospital, Capital Medical University, Beijing, China; 3 Department of Neurology, the First Affiliated Hospital of College of Medicine, Zhejiang University, Hangzhou, China; 4 Department of Neurology, the Daxing District Hospital of Beijing, Beijing, China; University Hospital-Eppendorf, Germany

## Abstract

**Objectives:**

To evaluate the safety of low-dose aspirin plus clopidogrel versus high-dose aspirin plus clopidogrel in prevention of vascular risk within 90 days of duration of dual antiplatelet therapy in patients treated with intracranial endovascular treatment.

**Methods:**

From January 2012 to December 2013, this prospective and observational study enrolled 370 patients with symptomatic intracranial atherosclerotic stenosis of ≥70% with poor collateral undergoing intracranial endovascular treatment. Antiplatelet therapy consists of aspirin, at a low-dose of 100 mg or high-dose of 300 mg daily; clopidogrel, at a dose of 75 mg daily for 5 days before endovascular treatment. The dual antiplatelet therapy continued for 90 days after intervention. The study endpoints include acute thrombosis, subacute thrombosis, stroke or death within 90 days after intervention.

**Results:**

Two hundred and seventy three patients received low-dose aspirin plus clopidogrel and 97 patients received high-dose aspirin plus clopidogrel before intracranial endovascular treatment. Within 90 days after intervention, there were 4 patients (1.5%) with acute thrombosis, 5 patients (1.8%) with subacute thrombosis, 17 patients (6.2%) with stroke, and 2 death (0.7%) in low-dose aspirin group, compared with no patient (0%) with acute thrombosis, 2 patient (2.1%) with subacute thrombosis, 6 patients (6.2%) with stroke, and 2 death (2.1%) in high-dose aspirin group, and there were no significant difference in all study endpoints between two groups.

**Conclusion:**

Low-dose aspirin plus clopidogrel is comparative in safety with high-dose aspirin plus clopidogrel within 90 days of duration of dual antiplatelet therapy in patients treated with intracranial endovascular treatment.

## Introduction

Intracranial atherosclerotic stenosis is one of the most common causes of ischaemic stroke worldwide [1]. Symptomatic intracranial atherosclerotic stenosis of ≥70% is associated with a high risk of recurrent stroke despite aggressive medical therapy [2], [3], [4]. As a means of preventing recurrent stroke, endovascular treatment remain to be considered potentially beneficial for patients with severe intracranial stenosis with insufficient collateral or with vulnerable plaque [5], [6], [7].

Aspirin in combination with clopidogrel preventing major thrombotic events has been the standard of care in patients undergoing intracranial endovascular treatment for more than a decade [8]. Despite its universal use, the optimal dose of aspirin from an efficacy and safety perspective remains unclear [9], [10]. Based on the studies for patients who underwent percutaneous coronary intervention, high-dose aspirin (≥300 mg daily) did not differ significantly from low-dose aspirin (75–100 mg daily) in prevention of cardiovascular death, myocardial infarction, or stroke, and stent thrombosis [11], [12], [13]. Given the high mortality of intracranial bleeding in intracranial endovascular treatment [14], [15], a low-dose aspirin strategy is subsequently adopted when using low-dose aspirin is no harm compared with high-dose aspirin in prevention of periprocedural complications and major vascular events in duration of dual antiplatelet therapy. So we perform a prospective study to evaluate the effect and safety of low-dose aspirin plus standard-dose clopidogrel versus high-dose aspirin plus standard-dose clopidogrel in prevention of vascular risk in patients with symptomatic intracranial atherosclerotic stenosis of ≥70% with poor collateral undergoing intracranial endovascular treatment within 90 days of duration of dual antiplatelet therapy after intervention.

## Methods

### Standard protocol approval and patient consent

The protocol for this prospective, observational, single-center study was approved by the institutional ethics committee at Beijing Tiantan Hospital before screening any patients. The written informed consent was obtained from all patients participating in the study.

### Patients

Patients were enrolled in this study according to the following criteria: 1. Primary or recurrent ischaemic stroke or transient ischaemic attack (TIA) in the target intracranial arterial territory within 90 days during the treatment with at least one antiplatelet drug. 2. Intracranial stenosis≥70%, lesion length<15 mm, target artery diameter≥2 mm, and a normal distal vessel bed on digital subtraction angiography (DSA). 3. No evidence of cardioembolism, including atrial fibrillation or recent myocardial infarction within one month. 4. Age≥30 years. 5. Two or more atherosclerotic risk factors including hypertension, hypercholesterolemia, diabetes mellitus, cigarette smoking, and obesity. Hypertension was defined as systolic blood pressure≥140 mmHg, diastolic blood pressure≥90 mmHg, or the patient is currently on an antihypertensive drug. Hypercholesterolemia was defined as a total cholesterol level ≥240 mg/dL or a low-density lipoprotein cholesterol level ≥160 mg/dL or current medication use for lowering the blood cholesterol level. Patients who used antidiabetic medications (insulin or oral hypoglycemics) were considered to have diabetes mellitus. Patients who smoked in the past or currently smoke were considered to have cigarette smoking history. Obesity was defined as a body mass index greater than 30 kg/m^2^. 6. Poor collateral was determined by the following three methods: ASITN/SIR Collateral Flow Grading System score<3 confirmed by DSA [16]; ≥30% decrease in cerebral blood flow in the territory distal to the target lesion by computed tomography (CT) perfusion (The reference area for CT perfusion is the contralateral hemisphere for anterior circulation lesions and anterior circulation territory for posterior circulation lesions) [17]; hemodynamic ischaemic lesion by magnetic resonance imaging (MRI) or CT.

Patients with the following conditions were excluded: nonatherosclerosis vasculopathy such as vasculitis and arterial dissection, diagnosed by comprehensive laboratory work (such as erythrocyte sedimentation rate or C-reactive protein elevations, antinuclear antibody, or antiphospholipid antibody positivity), vascular imaging, and clinical evaluation.

Using a traditional clinical definition of ischaemic events, we defined ischaemic stroke as a new focal neurologic deficit of sudden onset lasting ≥24 hours with lesion detected by CT or MRI and not caused by hemorrhage and TIA was defined as acute onset of a focal neurologic deficit lasting <24 hours.

From January 2012 to December 2013, 370 consecutive patients with symptomatic ICAS ≥70% on vascular imaging were enrolled.

### Medical treatment before procedure

All the enrolled patients received medical treatment. The dose of aspirin was used same as the dose recommended by the doctors in emergency or out-patient department. Other medical treatment consists of clopidogrel, at a dose of 75 mg daily for 5 days before procedure; and management of the atherosclerotic risk factors including elevated systolic blood pressure and elevated low-density lipoprotein cholesterol levels, and diabetes. Patients who have not been on dual antiplatelet therapy for five days prior to procedure are given a 300 mg loading dose of clopidogrel between 6 and 24 hours before the procedure.

### Endovascular treatment protocol

The procedure was performed by experienced neurointerventionists, who had each done at least 100 endovascular procedures for intracranial atherosclerotic stenosis. Intravenous heparin was administered after placement of a 6-Fr sheath or a 5-Fr sheath by transfemoral artery or transradial artery (only for posterior circulation and tortuous arch) as a bolus (75 U/kg) followed by half the dose one hour later, and if the procedure last longer than 2 hours, a quarter of the initial dose was given at every hour thereafter. The guiding catheter was advanced into the cervical vertebral or internal carotid artery as high as the vessel tortuosity allowed.

Device selection depended on arterial access and lesion morphology. For patients with smooth arterial access and Mori A lesion [18], the Apollo balloon-mounted stent (MicroPort, Shanghai, China) was selected. For patients with tortuous arterial access and Mori B or C lesion, or lesion with a significant mismatch in the diameter between proximal and distal segment, angioplasty plus self-expanding stent (Gateway balloon plus Wingspan stent system [Styker, Maple Grove, Minnesota, USA]) is preferred. For patients with tortuous arterial access with a Mori A lesion, or small target vessel diameter (<2.5 mm), direct dilation with Gateway balloon was selected. If severe dissection or elastic recoil occurred after angioplasty, a balloon-mounted stent (for patients with less tortuous access) or Wingspan (for patients with severe tortuous access or small target vessel) stent were allowed to be implanted. Technical success rate of angioplasty was defined as complete coverage of the target lesion with the residual stent stenosis <50% and with thrombolysis in cerebral ischaemia (TICI) grade 3 [16]. Intraprocedural blood pressure was monitored every 5 minutes and postprocedural blood pressure was monitored from hourly to once every 4 hours for at least 1 day, and systolic blood pressure was kept between 100 and 120 mm Hg.

### Medical management after procedure

After intervention, all patients were given a weight-based dose of 0.4 to 0.6 mL low molecular weight heparin every 12 hours subcutaneously for 3 days and monitored closely until discharge. Dual antiplatelet therapy consists of aspirin, at a dose same as the pre-procedural regimen, clopidogrel, at a dose of 75 mg daily, for 90 days after procedure. After 90 days, 100 mg aspirin or 75 mg clopidogrel daily is continuously used. Other medical therapies in follow-up are management of the atherosclerotic risk factors including elevated systolic blood pressure and elevated low-density lipoprotein cholesterol levels, diabetes, smoking, excess weight, and insufficient exercise.

### Study endpoints

During the period of follow-up, patients were contacted monthly by phone to determine whether any events had occurred. At the first month after intervention and then every three months, patients were examined face-to-face by two neurologists. The study endpoints within 90 days below were collected. The study endpoint was acute thrombosis, subacute thrombosis, stroke (in any vascular territory) or death, non-stroke hemorrhage, and hyperperfusion symptoms within 30 days after intervention and stroke in the qualifying artery territory or death, and non-stroke hemorrhage beyond 30 days after intervention. Acute thrombosis was defined as thrombosis at 0 to 24 hours after intervention; Subacute thrombosis was defined as thrombosis at 24 hours to 30 days after intervention. All subacute thrombosis was confirmed by an emergency DSA. Stroke was defined as a new focal neurological deficit of sudden onset that lasted ≥24 hours. If a stroke is suspected during the follow-up period, the patient is examined by a neurologist, and the ischaemic or hemorrhagic stroke was diagnosed by brain MRI or CT. Non-stroke hemorrhage is defined as any subdural or epidural hemorrhage or a systemic hemorrhage. Hyperperfusion symptoms were defined as headache, seizure, delirium, or a neurologic deficit combined with a 100% increase of velocity by Transcranial Doppler compared with baseline [19].

### Statistical Analysis

Continuous variables were presented as means±SD. Categorical variables were presented as percentages. Student's *t*-test or the Mann-Whitney U-test (when continuous variables had skewed distributions) was used to identify the difference in each of the variables between patients with low-dose aspirin and high-dose aspirin. The difference in each of the categorical variables between the two groups was tested with χ^2^ or Fisher's exact tests (when the expected cell frequency was <5). A two-tailed P value less than 0.05 was considered statistically significant. All analyses were performed using the software SAS 9.2 (SAS Institute Inc., Cary, NC, USA).

## Results

### Patient Characteristics

Among 370 patients (mean age 59 years, range 37–80 years), 273 patients received aspirin at a low-dose of 100 mg and clopidogrel at a dose of 75 mg and 97 patients received aspirin at a high-dose of 300 mg and clopidogrel at a dose of 75 mg. The data on study endpoints presented below are based on all adverse events as of April 11, 2014, when the last patient enrolled completed the 90-day evaluation. The mean follow-up was 15 months (range from 1–28 months). Among all the patients, 10 patients had loss of follow-up within 90-day evaluation.

There were no significant differences with respect to any of the baseline characteristics of the patients between the two groups (Table 1). In low-dose aspirin group, there were 159 (159/273, 58.2%) patients treated with balloon-mounted stent, 89 (89/273, 32.6%) patients treated with balloon angioplasty plus expendable stent, and 22 (22/273, 8.1%) patients treated with balloon angioplasty alone. In high-dose aspirin group, there were 63 (63/97, 64.9%) patients treated with balloon-mounted stent, 24 (24/97, 24.7%) patients treated with balloon angioplasty plus expendable stent, and 6 (6/97, 6.2%) patients treated with balloon angioplasty alone. The procedure failed in 3 (3/273, 1.0%) patients in low-dose aspirin group and 4 (4/97, 4.1%) patients in high-dose aspirin group. There were no significant differences between the two groups in endovascular methods (Table 2).

**Table 1:** Comparison of baseline characteristics of patients receiving low-dose aspirin and patients receiving high- dose aspirin*.

Characteristic	Patients receiving low-dose aspirin (N = 273)	Patients receiving high-dose aspirin (N = 97)	P value
**Age** **— yr**	59.2±8.1	58.2±9.3	0.39
**Male sex — no. (%)**	228(83.5)	75 (77.3)	0.22
**Risk factors— no. (%)**			
Hypertension	222 (81.3)	73 (75.3)	0.24
Hyperlipidemia	158 (57.9)	57 (58.8)	0.91
Diabetes mellitus	92 (33.7)	33 (34.0)	1.00
Smoking	164 (60.1)	47 (48.5)	0.07
Obesity	24 (8.8)	9 (9.3)	0.84
**Qualifying ischaemic events — no. (%)**			
TIA	149 (54.6)	64 (66.0)	0.06
Stroke	124 (45.4)	33 (34.0)	0.06
**Symptomatic qualifying artery — no. (%)**			
BA	109 (40.0)	38 (39.2)	1.00
Intracranial vertebral	101 (37.0)	35 (36.1)	0.90
MCA	34 (12.5)	15 (15.5)	0.49
Intracranial carotid	29 (10.6)	9 (9.3)	0.85
**Cases with loading dose of clopidogrel**	12 (4.4)	4 (4.1)	1.00
**Time from qualifying event to endovascular treatment— days**			0.52
Median	25	22	
Interquartile range	10–41	12–30	

*Plus–minus values are means ±SD. Baseline characteristics of the two groups were compared with the use of either an independent groups t-test (for means) or a chi-square test (for percentages). TIA denotes transient ischaemic attack; BA denotes basilar artery; MCA denotes middle cerebral artery.

**Table 2:** Comparison of endovascular treatment data and study endpoints within 30 days and 90 days after intervention of patients receiving low-dose aspirin and patients receiving high-dose aspirin*.

	Patients receiving low-dose aspirin (N = 273)	Patients receiving high-dose aspirin (N = 97)	P value
**Endovascular treatment methods — no. (%)**			
Balloon-mounted stenting	159 (58.2)	63 (64.9)	0.28
Balloon angioplasty + expendable stenting	89 (32.6)	24 (24.7)	0.16
Balloon angioplasty	22 (8.1)	6 (6.2)	0.66
Failure	3 (1.0)	4 (4.1)	0.08
**Multi vessels treated — no. (%)**	13 (4.8)	3 (3.1)	0.77
**Loss of follow-up — no. (%)**	6 (2.2)	4 (4.1)	0.30
**Dual Antiplatelet drugs use within 90 days — no. (%)**	270 (98.9)	96 (98.9)	1.00
**Study endpoints within 30 days — no. (%)**			
Acute thrombosis	4 (1.5)	0 (0)	0.58
Subacute thrombosis	5 (1.8)	2 (2.1)	1.00
Thrombosis	9 (3.3)	2 (2.1)	0.74
Ischaemic stroke	10(3.7)	4 (4.1)	0.77
Hemorrhagic stroke	3 (1.1)	1 (1.0)	1.00
Death	1 (0.4)	2 (2.1)	0.17
Non-stroke hemorrhage	1(0.4)	1 (1.0)	0.46
Hyperperfusion symptoms	9 (3.3)	5 (5.2)	0.54
**Study endpoints within 90 days — no. (%)**			
Ischaemic stroke	14 (5.1)	5 (5.2)	1.00
Hemorrhagic stroke	3 (1.1)	1 (1.0)	1.00
Death	2 (0.7)	2 (2.1)	0.28
Non-stroke hemorrhage	2 (0.7)	1 (1.0)	1.00

*Plus–minus values are means ±SD. Baseline characteristics of the two groups were compared with the use of either an independent groups t-test (for means) or a chi-square test (for percentages).

### Study endpoints

Within 30 days after intervention, there were 4 patients (4/273, 1.5%) with acute thrombosis and 5 patients (5/273, 1.8%) with subacute thrombosis compared with no patient (0/97, 0%) with acute thrombosis and 2 patient (2/97, 2.1%) with subacute thrombosis in high-dose aspirin group. There were 10 patients (10/273, 3.7%) with ischaemic stroke including 4 patients due to subacute thrombosis, 3 patients due to perforator infarction, 2 patients due to acute thrombosis, 1 patients due to embolization, in low-dose aspirin group, compared with 4 patients (2/97, 4.1%) including 2 patients due to perforator infarction and 2 patient due to subacute thrombosis in high-dose aspirin group. There were 3 patient (3/273, 1.1%) with hemorrhagic stroke including 2 patients due to guidewire perforation and 1 patient due to a delayed intraparenchymal hemorrhage related to hyperperfusion, compared with 1 patient (1/97, 1.0%) with a parenchymal hemorrhage out of the territory of qualifying artery in high-dose aspirin group. There were 1 death (1/273, 0.4%) due to subacute thrombosis in low-dose aspirin group, compared with 2 death (2/97, 2.1%) due to a delayed intraparenchymal hemorrhage related to hyperperfusion in high-dose aspirin group. There were 1 patient (1/273, 0.4%) with aural hemorrhage without requiring blood transfusion and surgery in low-dose aspirin group, compared with 1 patient (1/97, 1.0%) with urine hemorrhage without blood transfusion and surgery in high-dose aspirin group. Among all the patients, 14 patients (14/370, 3.7%) of with hyperperfusion symptoms was observed, including 9 patients in low-dose aspirin group in which 1 patient (1/9, 11%) evolved to hemorrhagic stroke, and 5 patients in high-dose aspirin group in which 3 patients (3/5, 60%) evolved to hemorrhagic stroke or death.

During the period of 30 days to 90 days after intervention, there were 4 (4/273, 1.5%) patients with ischaemic stroke due to in-stent restenosis, 1 non-stroke death (1/273, 0.4%) due to pneumonia and 1 patient (1/273, 0.4%) with nasal hemorrhage without requiring blood transfusion and surgery in low-dose aspirin group, and there was 1 patient (1/97, 1.0%) with ischaemic stroke due to in-stent restenosis in high-dose aspirin group. There were no significant differences in all study endpoints between two groups (Table 2).

All 11 patients with thrombosis were treated with a 10–20 mg dose of alteplase (Boehringer Ingelheim, Ingelheim, Germany) mixed as 1 mg per milliliter and/or a 6–10 ml tirofiban hydrochloride (Wuhan Grand Pharma, Wuhan, China) injected via guide catheter. There 2 patients with acute thrombosis remaining asymptomatic after the intervention and 1 death due to failure of intervention.

## Discussion

To prevent periprocedural platelet emboli and thrombus formation, patients are typically treated for a variable number of days before the intracranial endovascular treatment with a combination of aspirin and clopidogrel [11], [12], [13]. The dose of each antiplatelet agent varies from operator to operator, and there is no consensus on the safety, dosage, or drug combination [11], [12], [13]. Particularly, the dose of aspirin varies greatly worldwide and it is given between 81 and 325 mg daily before the procedure [10]. This variation in practice is partly due to the fact that no comparisons of aspirin dose have been done in intracranial endovascular treatment since its routine use a decade ago. Based on the studies on percutaneous coronary intervention suggested high-dose aspirin did not differ significantly from low-dose aspirin in prevention of periprocedural complications [13], we want to evaluate wither there is the difference in safety and efficacy of the low-dose of aspirin plus standard-dose clopidogrel versus high-dose of aspirin plus standard-dose clopidogrel in intracranial endovascular treatment within the duration of dual antiplatelet therapy.

Our study suggested that in patients with severe symptomatic intracranial atherosclerotic stenosis with poor collateral, low-dose aspirin (100 mg daily) does not increase risk of ischaemic events compared with high-dose aspirin (300 mg daily). The risk of hemorrhagic stroke or death evolving from hyperperfusion symptom (3/5, 60%) in high-dose aspirin group may be higher than that (1/9, 11%) in low-dose aspirin group. Considering an incidence of hyperperfusion symptoms was 3.7% (14/370) observed in this cohort with poor collateral, this finding may suggest that low-dose aspirin plus clopidogrel may be a reasonable option for intracranial endovascular treatment in patients with poor collateral.

The overall complications rate within 30 days after intervention in this study was lower than that reported in in the SAMMPRIS trial [4]. Aside from the different antiplatelet regimen, other factors are the following: 1.This study was performed a large volume stroke center. 2. The procedures were performed by operators with vast experience of intracranial endovascular treatment, who have long pasted the steep portion of the learning curve [20]. 3. A tailored endovascular treatment method was selected relying on individual access and lesion anatomy and it may be safe than using a single endovascular device for intracranial stenosis [21]. 4. The time from the last episodic event to the procedure was substantially longer than that in SAMMPRIS. The longer waiting time may have allowed the plaque to stabilize and thrombus to dissolve, and probably reduced the risk of perforator infarction by snow plow effect during the procedure as well. 5. The difference of baseline characteristics may cause bias. This study only studied the Asia population. The rates of hypertension, hyperlipidemia, and diabetes mellitus except smoking are slightly lower than that in the SAMMPRIS study. The majority of qualifying event was TIA and the symptomatic qualifying artery was BA compared these was stroke and MCA in the SAMMRPIS study [4]. However, Concerns have been raised to these explanations because the event rates in the registry are usually lower than randomized trial.

In this study, the subacute thrombosis rate in low-dose aspirin is 1.8% (5/273) which is low than 12.1% (4/33) in a previous study using 100 mg aspirin plus 75 mg clopidogrel 3 days before the procedure [22]. We thought that a 5 days dual antiplatelet regimen may be more efficacious than a 3 days dual antiplatelet regimen in achieving high levels of platelet inhibition.

In addition, the ischemic stroke within 30 days after intervention was ascribed to thrombosis and perforator stroke, compared that during the period of 30–90 days was due to in-stent restenosis. The high occurrence of thrombosis was different that observed in the SAMMPRIS study but similar with that found in a previous study on coronary artery disease [14], [23].

Our study showed the risk of bleeding with dual antiplatelet therapy within 90 days after intervention is low. The overall non-stroke hemorrhage rate within 90 days after intervention was 0.8% (3/370) which is comparative with a previous study in which the incidence of minor bleeding events of patients with a 3-month dual antiplatelet therapy after implantation of zotarolimus-eluting stent for coronary artery disease was 1.5% [24].

There some limitations in this study. First, the sample size is small. Secondly, the dose of aspirins was not determined by randomization. Thirdly, this study did not focus on the mechanism of complications and the issues about antiplatelet drugs resistance or the effectiveness of the antiplatelet therapy by using lab tests. We try to improve these in the future study.

In summary, antiplatelet therapy consisting of low-dose aspirin plus clopidogrel is comparative in safety with high-dose aspirin plus clopidogrel for patients with severe symptomatic intracranial atherosclerotic stenosis with poor collateral undergoing intracranial endovascular treatment.
